# Transcriptomics of Maternal and Fetal Membranes Can Discriminate between Gestational-Age Matched Preterm Neonates with and without Cognitive Impairment Diagnosed at 18–24 Months

**DOI:** 10.1371/journal.pone.0118573

**Published:** 2015-03-30

**Authors:** Athina Pappas, Tinnakorn Chaiworapongsa, Roberto Romero, Steven J. Korzeniewski, Josef C. Cortez, Gaurav Bhatti, Nardhy Gomez-Lopez, Sonia S. Hassan, Seetha Shankaran, Adi L. Tarca

**Affiliations:** 1 Perinatology Research Branch, Eunice Kennedy Shriver National Institute of Child Health and Human Development / NIH / DHHS, Bethesda, MD and Detroit, MI, United States of America; 2 Department of Pediatrics, Division of Neonatal and Perinatal Medicine, Wayne State University, Detroit, MI, United States of America; 3 Department of Obstetrics and Gynecology, Wayne State University, Detroit, MI, United States of America; 4 Department of Obstetrics and Gynecology, University of Michigan, Ann Arbor, MI, United States of America; 5 Department of Epidemiology and Biostatistics, Michigan State University, East Lansing, MI, United States of America; 6 Department of Immunology and Microbiology, Wayne State University, Detroit, MI, United States of America; The George Washington University, UNITED STATES

## Abstract

**Background:**

Neurocognitive impairment among children born preterm may arise from complex interactions between genes and the intra-uterine environment.

**Objectives:**

(**1**) To characterize the transcriptomic profiles of chorioamniotic membranes in preterm neonates with and without neurocognitive impairment via microarrays and (**2**) to determine if neonates with neurocognitive impairment can be identified at birth.

**Materials/Methods:**

A retrospective case-control study was conducted to examine the chorioamniotic transcriptome of gestational-age matched very preterm neonates with and without neurocognitive impairment at 18–24 months’ corrected-age defined by a Bayley-III Cognitive Composite Score <80 (n = 14 each). Pathway analysis with down-weighting of overlapping genes (**PADOG**) was performed to identify KEGG pathways relevant to the phenotype. Select differentially expressed genes were profiled using qRT-PCR and a multi-gene disease prediction model was developed using linear discriminant analysis. The model’s predictive performance was tested on a new set of cases and controls (n = 19 each).

**Results:**

**1**) 117 genes were differentially expressed among neonates with and without subsequent neurocognitive impairment (p<0.05 and fold change >1.5); **2**) Gene ontology analysis indicated enrichment of 19 biological processes and 3 molecular functions; **3**)**PADOG** identified 4 significantly perturbed KEGG pathways: oxidative phosphorylation, Parkinson’s disease, Alzheimer’s disease and Huntington’s disease (q-value <0.1); **4**) 48 of 90 selected differentially expressed genes were confirmed by qRT-PCR, including genes implicated in energy metabolism, neuronal signaling, vascular permeability and response to injury (e.g., up-regulation of *SEPP1*, *APOE*, *DAB2*, *CD163*, *CXCL12*, *VWF;* down-regulation of *HAND1*, *OSR1*)(p<0.05); and **5**) a multi-gene model predicted 18–24 month neurocognitive impairment (using the ratios of *OSR1/VWF* and *HAND1/VWF* at birth) in a larger, independent set (sensitivity = 74%, at specificity = 83%).

**Conclusions:**

Gene expression patterns in the chorioamniotic membranes link neurocognitive impairment in preterm infants to neurodegenerative disease pathways and might be used to predict neurocognitive impairment. Further prospective studies are needed.

## Introduction

While advances in perinatal medicine have improved the survival and short-term outcomes of preterm neonates, rates of neurodevelopmental impairment at 18–24 month follow-up and beyond remain high [1–7]. Neurocognitive deficits are among the most prevalent and most debilitating forms of early childhood disabilities, reported in 23% of infants born 27–32 weeks’ gestation and 37% of infants born at 22–26 weeks’ gestation [4]. Cognitive impairment can impact adaptive functioning, conceptual, social, and practical domains, and lead to high personal, familial, societal and financial costs. The estimated US average lifetime costs to care for an individual with intellectual impairment is $1,014,000 [8].

Neurocognitive disorders may arise from complex interactions between genes and the environment, originating prior to birth. Though postnatal interventions have afforded limited success in preventing neurocognitive and developmental impairments associated with prematurity, prenatal interventions such as antenatal steroids [9–13] and magnesium sulfate [14–18] provide greater population impact. The search for intrauterine or perinatal disease pathways associated with fetal and neonatal brain injury may afford new insights into preventive measures and disease pathogenesis. Other investigators have utilized mRNA levels in blood samples collected soon after birth to identify children at risk for other neurodevelopmental disorders such as cerebral palsy [19] and autism [20].

The fetal membranes are an alternative source of fetal DNA and of human fetal stem cells [21] that may be impacted by intrauterine stimuli. Stem cells derived from the fetal membranes are available after every preterm birth and have pluripotent differentiation potential [22, 23]. Embryonic [24, 25] and pluripotent stem cells [26] have emerged as powerful tools in the study of normal neuronal development and of neuropsychiatric disorders such as Parkinson’s disease [27–30], Rett syndrome [31–33], fragile X [34, 35], Down’s syndrome [36, 37] and schizophrenia [38–41]. Recent data suggests that there are no significant differences between human embryonic and induced pluripotent stem cell gene expression levels [42–44], thus the study of pluripotent stem cells (including fetal amnion and chorion cells) [21] provides a pragmatic, yet noncontroversial methodology to readily access large numbers of relevant cells from multiple cases and controls. Changes in gene expression of the chorioamniotic membranes may capture in-utero insults and fetal response to injury in preterm infants. Our objectives were (1) to characterize the molecular profile of the chorioamniotic membranes of preterm neonates with and without neurocognitive impairment at 18–24 months’ corrected age and (2) to determine if neonates with neurocognitive impairment have a molecular signature that can be used to predict future disease onset at the time of birth.

## Materials and Methods

### Study participants

A retrospective case-control study was conducted to examine the chorioamniotic membranes of 66 very preterm neonates with and without neurocognitive impairment. Cases and controls were singleton neonates born at Hutzel Women’s Hospital (Detroit, MI) between January 1, 2006 and December 31, 2010 who were matched for gestational age (+ 2 weeks) and born between 23 and 32 weeks of gestation. Neurocognitive impairment was defined by a Bayley scales of infant development, 3^rd^ Edition cognitive composite score <80 with or without associated neuromotor impairment at 18–24 months’ corrected age [45]. Control infants had normal neurodevelopmental assessments including cognitive testing, neurological examination and gross motor function [46]. The Bayley scales of infant development, 3^rd^ Edition has a mean (SD) cognitive composite score of 100 (15). A cut score of 80 was selected based on data from recent population studies [47, 48]. The infants’ mothers provided written informed consent for the collection of biological materials and clinical data for research purposes under protocols approved by the Institutional Review Boards of the *Eunice Kennedy Shriver* National Institute of Child Health and Human Development (NICHD/NIH/DHHS, Bethesda, Maryland) and the Human Investigation Committee of Wayne State University (Detroit, MI, USA). Neonatal and neurodevelopmental outcomes were abstracted from the clinical records.

### Sample collection

Chorioamniotic membrane samples were retrieved from the bank of biologic samples of Wayne State University, the Detroit Medical Center, and the Perinatology Research Branch of the *Eunice Kennedy Shriver* National Institutes of Child Health and Human Development (NICHD) (Detroit, MI). At the time of specimen collection, the fetal membranes were dissected from the placenta, rolled, cut into small pieces and flash-frozen using liquid nitrogen [49]. In addition, a section of membranes containing maternal decidua was fixed and embedded in paraffin. 5mm paraffin sections were stained with hematoxylin and eosin and examined under bright-field light microscopy [50]. Histological examinations were reported by placental pathologists who were blinded to the group assignment and all clinical information.

### RNA isolation

Total RNA was isolated from snap-frozen tissues using TRIzol reagent (Invitrogen, Carlsbad, CA, USA) and Qiagen RNeasy kit (Qiagen, Valencia, CA, USA) according to the manufacturers’ recommendations. RNA concentrations and A260nm/A280nm ratios were assessed using a NanoDrop 1000 (Thermo Scientific, Wilmington, DE, USA). The 28S/18S ratios and RNA integrity numbers were assessed using a Bioanalyzer 2100 (Agilent Technologies, Wilmington, DE, USA).

### Microarray experiment

Total RNA (500 ng) was amplified and biotin-labeled with the Illumina TotalPrep RNA Amplification Kit (Ambion, Austin, TX, USA). Labeled cRNAs were hybridized to Illumina’s HumanHT-12 Expression BeadChip (Illumina, San Diego, CA, USA). BeadChips were imaged using a BeadArray Reader, and raw data were obtained with BeadStudio Software v3.2.7 (Illumina). Raw and preprocessed data were deposited in the Gene Expression Omnibus[51] at NCBI (reviewer access link: http://www.ncbi.nlm.nih.gov/geo/query/acc.cgi?token=otytuigsnvutlyj&acc=GSE61822).

### qRT-PCR assays with biomark system

Total RNA (500 ng) was reverse transcribed using the SuperScript III First-Strand Synthesis System and oligo(dT) 20 primers (Invitrogen, Carlsbad, CA, USA). TaqMan Assays (Applied Biosystems, Foster City, CA, USA) were used for gene expression profiling on the Biomark high-throughput qRT-PCR system (Fluidigm, San Francisco, CA, USA) according to the manufacturers’ instructions. Briefly, a 0.2X pool of specific gene expression assays (S1 Table) (Applied Biosystems) was used as the source of primers. Preamplification reactions contained 1.25μl cDNA, 2.5 μl TaqMan PreAmp master mix (Applied Biosystems) and 1.25μl pooled assay mix. The reaction was performed with a thermal cycler for 14 cycles at 95°C for 15 seconds and 60°C for 4 minutes. After cycling, the reaction was diluted 1:5 with ddH_2_O to a final volume of 25μl. Next, a Fluidigm 96.96 Dynamic Array chip was primed in an Integrated Fluidic Circuit controller. Then, 2.5μl 20X TaqMan gene expression assays (Applied Biosystems) were mixed with 2.5μl 2X assay loading reagent (Fluidigm) and loaded into the assay inlet on the 96.96 array chip. 2.25μl preamplified cDNA was mixed with 2.5μl TaqMan Universal PCR master mix (Applied Biosystems) and 0.25μl 20X sample loading reagent (Fluidigm), and loaded into the sample inlet on the chip. The chip was returned to the Integrated Fluidic Circuit controller for loading. After loading the samples and assays, the chip was placed into the Biomark System to run the reactions.

### Statistical analysis

*Clinical Data*: The maternal and neonatal demographic and clinical characteristics of the two study groups were compared using the Wilcoxon rank-sum test or t-test for between-group comparisons of continuous data as appropriate. The Chi-square or Fisher’s exact tests were used for comparisons of categorical data. Statistical analyses of demographic data were performed using SPSS version 19 (SPSS Inc, Chicago, IL). A p-value <0.05 was used to designate statistical significance.

*Microarray Data*: Gene expression data that was measured on the first set of cases and controls (n = 14 each) was offset by adding a constant value of 1 to enable subsequent log (base 2) transformation. A quantile-normalization procedure [52] implemented in the *preprocessCore* package of *Bioconductor* (http://www.bioconductor.org) was then applied to remove non-biological systematic biases in the intensity data and hence make it comparable between arrays. A linear model was fit to the data of each probe to test the association between the gene expression and the phenotype (neurocognitive impairment vs. control). The significance of the group coefficient in the linear model was inferred using p<0.05 from a paired moderated t-test together with a minimum of a 1.5-fold-change between groups [53]. Although it is customary in microarray analyses to choose significant genes based on adjusted p-values [54], as shown in the MicroArray Quality Control (MAQC) study [55], reproducible differential expression results also can be obtained using a nominal p<0.05 cut-off provided the magnitude of changes is considered in gene selection. Since the microarray study was followed by a targeted qRT-PCR experiment involving a new set of samples that could rule out some of the eventual false positives, we used a less stringent significance cut-off in the microarray experiment to minimize false negatives. Gene Ontology analysis of significant genes was conducted using *GOstats* [56]. Pathway analysis with down-weighting of overlapping genes (PADOG) was conducted to identify Kyoto Encyclopedia of Genes and Genomes (KEGG) gene sets and biological pathways relevant to the group phenotype [57, 58]. PADOG leverages differential evidence from all genes in a pathway while giving more weight to genes that are specific to a given pathway than to those that appear in multiple pathways.

*qRT-PCR Data*: Ninety differentially expressed genes based on the microarray data were selected for qRT-PCR profiling in an extended set of cases and controls (n = 33 each). There were two goals with the qRT-PCR data analysis. First, to verify the 90 genes found significant based on microarray analysis in the first set of 14 case-control pairs, and second, to determine accuracy of a multi-gene predictor based on these data. For the first goal, the extended set of 33 case-control pairs were used to verify the microarray results. Differential expression based on Cycle threshold (Ct) data was inferred using p*<*0.05 from a one-tailed t-test using the direction of change for each of the 90 genes in the microarray experiment as the hypothesis test alternative.

For the second goal, qRT-PCR measured expression data were split into a “training” set and a “test” set (“hold-out validation” procedure). The training/learning set included 14 cases and 14 controls that were used in the microarray experiment. The test set included 19 cases and 19 controls with blinded class membership, not used in any stage of the prediction model development. To obtain a classifier from the training data without using any information from the test data, all 90 candidate genes found significant by microarrays were considered as inputs in a classifier development pipeline that we have previously described in detail [59] and adapted for the qRT-PCR data in the current study. Briefly, with this procedure, each of the 90 genes was considered in turn as a potential normalizer and log_2_ gene expression ratios between each remaining gene and the normalizer gene were computed. The log_2_ ratios were simply the differences in –Ct values of each gene and the reference gene. The gene expression ratios were then ranked using p-values from a moderated t-test and those that did not change at least 1.2 fold were discarded. The top ranked gene ratios were used in a linear discriminant analysis (LDA) model implemented in the MASS package in R (http://www.r-project.org/). The number of top ratios to use in the classifier was optimized by maximizing the average model sensitivity over three cut-offs of specificity (80, 85, and 90%). The sensitivity calculations were performed using a five time repeated three-fold cross validation procedure on the training data that included both the ratios ranking and LDA model fitting steps, functionality that we have made available in the maPredictDSC package [59] of Bioconductor (http://www.bioconductor.org). The sensitivity estimate of the resulting model for the optimal number of top ratios was determined for each possible normalizer gene, and the one that provided the highest sensitivity was retained. The ratios ranking and LDA model training were then performed on all training data to produce a final model. The cross-validated performance on the training set for the optimum number of predictors was determined for a quadratic discriminant analysis (QDA) and a support vector machines (SVM) classifier as well [60]. Implementations of these algorithms in R were available in the *MASS* and *e1071* packages, respectively.

**Fig 1:**
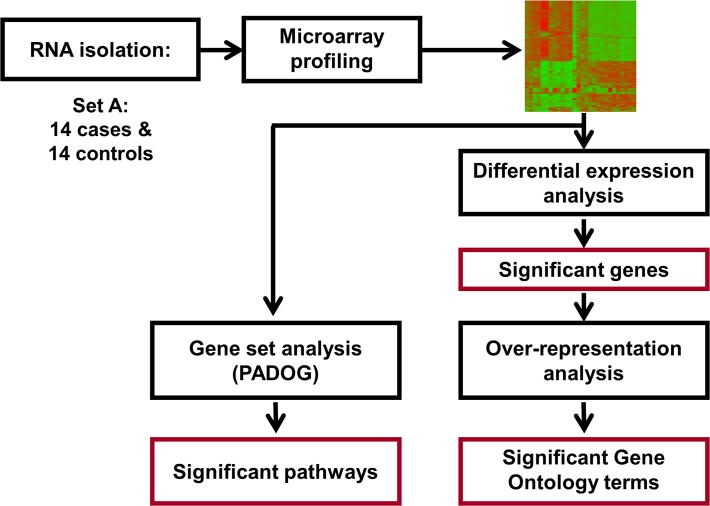
Microarray study flow diagram. The microarray analysis was performed on 14 cases and 14 controls (Set A) to identify significant genes, KEGG pathways and Gene Ontology terms.

**Fig 2:**
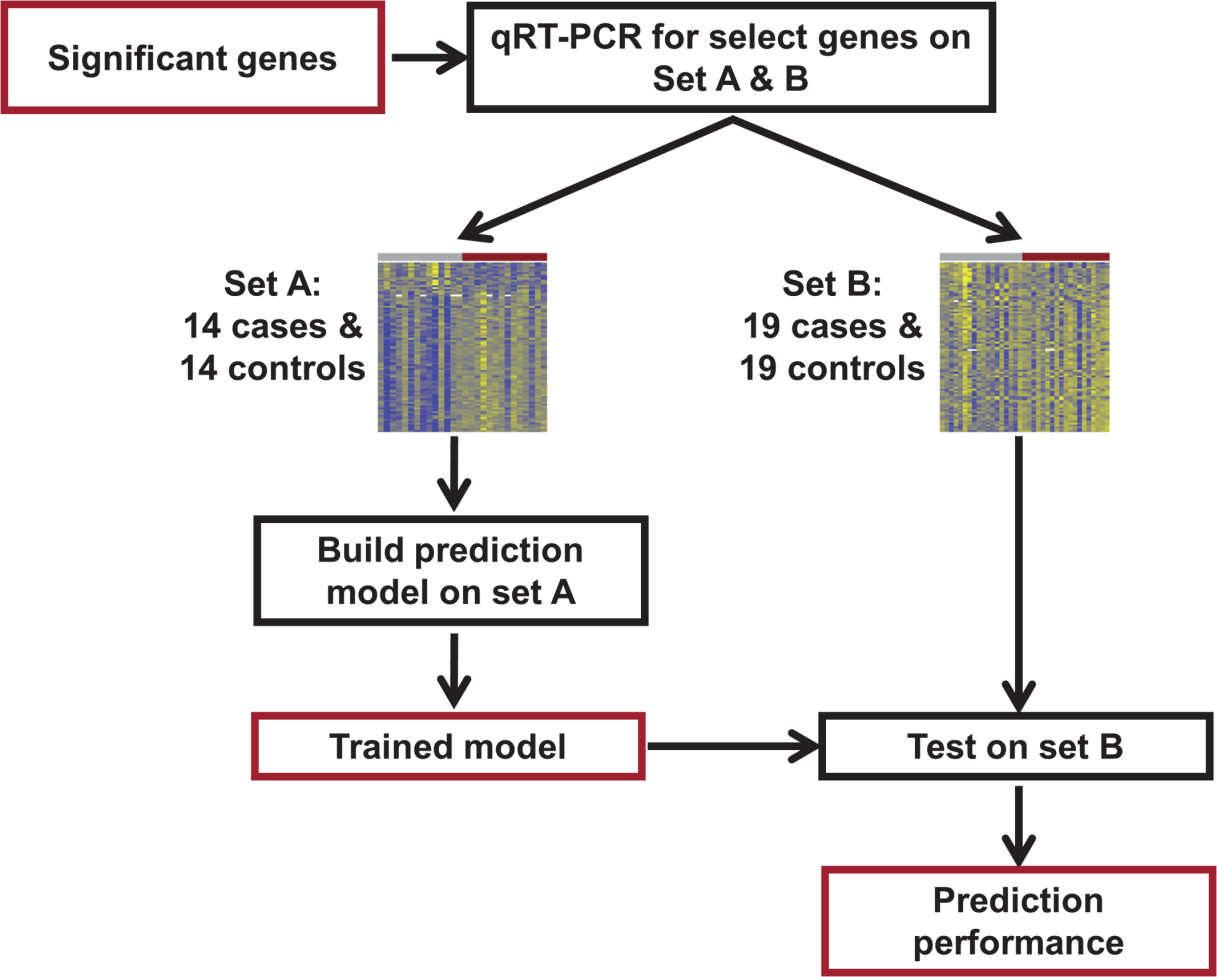
qRT-PCR study flow diagram. Differentially expressed genes identified from the microarray study were profiled using qRT-PCR for validation and construction of a multi-gene disease classifier using an extended set of 33 cases and 33 controls. Set A was used to build the prediction model and then this model was tested on set B, consisting of 19 cases and 19 controls.

The trained model was applied on the test set to calculate an unbiased estimate of the predictive performance (sensitivity at fixed specificity). The predictive performance of the gene based classifier was compared with that of a model that used clinical information available at the time of birth: gestational age, gender, small for gestational age status, 5-minute Apgar score, labor and chorioamnionitis. **Figs. 1** and **2** provide a schematic representation of the microarray and qRT-PCR study designs.

## Results

**Tables 1** and **2** display the maternal and neonatal demographic and clinical characteristics. There were no significant differences between cases and controls for the maternal variables assessed. Distributions of neonatal variables assessed at delivery also did not differ between groups. However, more neonates in the neurocognitive impairment group had evidence of brain injury on post-natal cranial ultrasonography (including severe periventricular-intraventricular hemorrhage, periventricular leukomalacia and ventriculomegaly), as expected. Median cognitive scores (±SD) were 70 ± 8.8 (range 54–75) in the neurocognitive impairment group and 90 ± 8.0 (range 85–115) in the control group. Eight children in the neurocognitive impairment group had cerebral palsy.

**Table 1:** Baseline maternal characteristics of the two study groups.

	Microarray	Study Groups		qRT-PCR	Study Groups	
Characteristic	Neurocognitive impairment group (CASES)	No impairment group (CONTROLS)	P-value*	Neurocognitive impairment group (CASES)	No impairment group (CONTROLS)	P-value*
	N = 14	N = 14		N = 19	N = 19	
Mother’s age, years- Mean (SD); Median (Q1, Q3)	26.7 (4.6); 25.5 (24.0, 31.2)	26.2 (3.5); 26.0 (25.0, 27.2)	NS	27.8 (5.1); 26.0 (25.0, 28.0)	26.1 (6.6); 25.0 (21.0, 30.0)	NS
*Race/ethnicity*,*(%)*						
Black	11/14 (78.5)	13/14 (92.8)	NS	17/19 (89.4)	15/19 (79.0)	NS
White	2/14 (14.3)	1/14 (7.1)	NS	2/19 (10.5)	2/19 (10.5)	NS
Other	1/14 (7.1)	0/14 (7.1)	NS	0/19 (0.0)	2/19 (10.5)	NS
*Insurance*, (%)						
Public	8/14 (57.1)	8/14 (57.1)	NS	15/18 (83.3)	13/19 (68.4)	NS
Private	5/14 (35.7)	5/14 (35.7)	NS	3/18 (16.7)	5/19 (26.3)	NS
None	1/14 (7.1)	1/14 (7.1)	NS	0/18 (0)	1/19 (5.3)	NS
*Maternal education*, (%)						
High school or less	5/14 (35.7)	7/14 (50.0)	NS	6/19 (31.6)	8/19 (42.1)	NS
More than high school	3/14 (21.4)	2/14 (14.3)	NS	2/19 (10.5)	3/19 (15.8)	NS
Unknown	6/14 (42.8)	5/14 (35.7)	NS	11/19 (57.9)	8/19 (42.1)	NS
Preeclampsia/HELLP/ HTN, (%)	5/14 (35.7)	4/14 (28.6)	NS	7/19 (36.8)	7/19 (36.8)	NS
pPROM, (%)	6/14 (42.8)	3/14 (21.4)	NS	5/19 (26.3)	3/19 (15.8)	NS
Preterm labor, (%)	3/14 (21.4)	6/14 (42.8)	NS	10/19 (52.6)	10/19 (52.6)	NS
Acute chorioamnionitis, (%)	7/14 (50.0)	7/14 (50.0)	NS	8/19 (57.9)	7/19 (36.8)	NS
Acute funisitis, (%)	7/14 (50.0)	5/14 (35.7)	NS	7/19 (36.8)	5/19 (26.3)	NS
Spontaneous labor / augmented labor, (%)	9/14 (64.3)	6/14 (42.8)	NS	1/19 (5.2)	0/19 (0.0)	NS
Induced labor / no labor, (%)	5/14 (35.7)	8/14 (57.1)	NS	9/19 (47.4)	10/19 (52.6)	NS
Antenatal steroids, (%)	14/14 (100)	14/14 (100)	NS	18/19 (94.7)	16/19 (88.9)	NS
Cesarean delivery, (%)	10/14 (71.4)	9/14 (64.3)	NS	14/19 (73.7)	13/19 (68.4)	NS

HTN- hypertension; HELLP- Hemolysis, elevated liver enzymes, low platelet count; pPROM- preterm premature rupture of membranes.

Insurance status was missing for 1 participant in the qRT-PCR experiment.

* P-value is significant at alpha < 0.05 level of significance.

**Table 2:** Baseline neonatal characteristics of the two study groups.

	Microarray	Study Groups		qRT-PCR	Study Groups	
Characteristic	Neurocognitive impairment group (CASES)	No impairment group (CONTROLS)	P-value*	Neurocognitive impairment group (CASES)	No impairment group (CONTROLS)	P-value*
	N = 14	N = 14		N = 19	N = 19	
Gestational age, week-Mean (SD); Median (Q1, Q3)	26.7 (1.6); 26.9 (25.2, 27.6)	26.6 (1.3); 26.5 (25.7, 27.0)	NS	26.6 (1.9); 26.3 (25.1, 27.7)	26.8 (1.8); 27.0 (25.1, 27.7)	NS
Birth weight, grams-Mean (SD); Median (Q1, Q3)	774.8 (158.6); 727.5(647.5, 888.0)	872.0 (172.9); 885.0 (755.2, 975.0)	NS	817.8 (139.0); 800.0 (700.0, 890.0)	851.8 (156.2); 849.0 (725.0, 935.0)	NS
Male, (%)	7/14 (50.0)	8/14 (57.1)	NS	14/19 (73.7)	13/19 (68.4)	NS
Small for gestational age at birth, (%)	4/14 (28.6)	1/14 (7.1)	NS	2/19 (10.5)	4/19 (21.1)	NS
*Apgar score < 3*, (%)						
At 1 minute	2/14 (14.3)	2/14 (14.3)	NS	5/19 (26.3)	4/19 (21.1)	NS
At 5 minutes	1/14 (7.1)	0/14 (0)	NS	0/19 (0.0)	0/19 (0)	NS
CRIB II score, Mean (SD); Median (Q1, Q3)	11 (3); 12 (8, 14)	11 (2); 10 (9, 12)	NS	11.4 (3.0); 11 (9, 13)	11.1 (2.6); 10.5 (9, 13.5)	NS
Respiratory distress syndrome, (%)	13/14 (92.8)	14/14 (100.0)	NS	19/19 (100)	17/19 (89.5)	NS
Bronchopulmonary dysplasia, (%)	10/14 (71.4)	7/14 (50.0)	NS	6/19 (31.6)	10/17 (58.8)	NS
Any intracranial hemorrhage, (%)	9/14 (64.3)	4/14 (28.6)	NS	8/19 (42.1)	10/19 (52.6)	NS
Severe intraventricular hemorrhage (Grade 3–4), (%)	5/14 (35.7)	2/14 (14.3)	NS	4/19 (21.1)	2/19 (10.5)	NS
Ventricular dilatation, (%)	5/14 (35.7)	2/14 (14.3)	NS	8/19 (42.1)	4/19 (21.1)	NS
Cerebellar hemorrhage, (%)	0/14 (0)	0/14 (0)	NS	2/19 (6.1)	0/19 (0)	NS
Cystic periventricular leukomalacia, (%)	1/14 (7.1)	0/14 (0)	NS	3/19 (15.8)	0/19 (0)	NS
Surgical necrotizing enterocolitis, (%)	0/14 (0)	0/14 (0)	NS	2/19 (10.5)	2/19 (10.5)	NS

CRIB II score- clinical risk index for babies score;

*P-value is significant at alpha < 0.05 level of significance

For all samples used in the microarray and qRT-PCR experiments, the 28S/18S ratios for RNA ranged from 1.7 to 2.0 and RNA integrity numbers ranged from 7.5 to 9.6.

### Microarray results

Differential expression analysis revealed moderate changes in the chorioamniotic membrane transcriptome of preterm neonates with and without neurocognitive impairment at 18–24 months: 133 probes corresponding to 117 unique genes were differentially expressed (p<0.05 and fold change >1.5) (**Tables 3** and **4**). Gene ontology analysis indicated enrichment of 19 biological processes (e.g., positive regulation of cell proliferation by VEGF-activated platelet derived growth factor receptor signaling pathway, etc.) and 3 molecular functions (cytokine binding, vascular-endothelial growth factor receptor activity and vascular-endothelial growth factor receptor binding) as shown in **Tables 5** and **6**. Pathway analysis with down-weighting of overlapping genes that uses information from all genes in a pathway to compute a pathway enrichment score, indicated four significant KEGG pathways: oxidative phosphorylation, Parkinson’s disease, Alzheimer’s disease and Huntington’s disease (q-value <0.1). Given the significant enrichment of biological processes, molecular functions and pathways identified, we selected genes involved in oxidative phosphorylation, mitochondrial function (central components of the aforementioned pathways) as well as other genes associated with neuronal development, signaling and response to injury for qRT-PCR validation.

**Table 3:** Overexpressed Illumina probes (N = 105) in the neurocognitive impairment group compared to the no impairment group.

Illumina Probe ID	ENTREZ^a^	SYMBOL^b^	Gene Name	Fold Change^c^	P-Value
ILMN_1768719	51109	RDH11	retinol dehydrogenase 11 (all-trans/9-cis/11-cis)	1.52	0.001
ILMN_1669409	11326	VSIG4	V-set and immunoglobulin domain containing 4	1.60	0.002
ILMN_3250257	94	ACVRL1	activin A receptor type II-like 1	2.26	0.004
ILMN_1765557	25903	OLFML2B	olfactomedin-like 2B	1.63	0.005
ILMN_1797731	64231	MS4A6A	membrane-spanning 4-domains, subfamily A, member 6A	1.79	0.006
ILMN_1689518	5175	PECAM1	platelet/endothelial cell adhesion molecule 1	1.51	0.006
ILMN_1701441	1902	LPAR1	lysophosphatidic acid receptor 1	1.52	0.007
ILMN_1728132	3945	LDHB	lactate dehydrogenase B	1.64	0.007
ILMN_2109416	256236	NAPSB	napsin B aspartic peptidase, pseudogene	1.82	0.008
ILMN_2060413	100133941	CD24	CD24 molecule	2.01	0.009
ILMN_1785071	6414	SEPP1	selenoprotein P, plasma, 1	2.58	0.009
ILMN_1805543	56999	ADAMTS9	ADAM metallopeptidase with thrombospondin type 1 motif, 9	1.60	0.010
ILMN_1745963	2350	FOLR2	folate receptor 2 (fetal)	1.53	0.010
ILMN_1678729	64374	SIL1	SIL1 homolog, endoplasmic reticulum chaperone (S. cerevisiae)	1.73	0.010
ILMN_1698019	5641	LGMN	legumain	1.81	0.010
ILMN_1668629	401115	C4orf48	chromosome 4 open reading frame 48	1.64	0.010
ILMN_2332964	5641	LGMN	legumain	1.69	0.011
ILMN_1685625	7351	UCP2	uncoupling protein 2 (mitochondrial, proton carrier)	1.50	0.012
ILMN_2359742	1508	CTSB	cathepsin B	1.69	0.012
ILMN_1763000	55803	ADAP2	ArfGAP with dual PH domains 2	1.52	0.012
ILMN_1662619	7035	TFPI	tissue factor pathway inhibitor (lipoprotein-associated coagulation inhibitor)	1.64	0.012
ILMN_2053103	30061	SLC40A1	solute carrier family 40 (iron-regulated transporter), member 1	2.46	0.013
ILMN_1694106	23171	GPD1L	glycerol-3-phosphate dehydrogenase 1-like	1.54	0.013
ILMN_1707124	7035	TFPI	tissue factor pathway inhibitor (lipoprotein-associated coagulation inhibitor)	1.75	0.013
ILMN_1668134	2944	GSTM1	glutathione S-transferase mu 1	1.53	0.014
ILMN_1761199	11309	SLCO2B1	solute carrier organic anion transporter family, member 2B1	1.61	0.014
ILMN_1689088	81035	COLEC12	collectin sub-family member 12	1.77	0.014
ILMN_2087656	11309	SLCO2B1	solute carrier organic anion transporter family, member 2B1	1.95	0.014
ILMN_1651262	3182	HNRNPAB	heterogeneous nuclear ribonucleoprotein A/B	1.53	0.015
ILMN_1764228	1601	DAB2	disabled homolog 2, mitogen-responsive phosphoprotein (Drosophila)	1.78	0.015
ILMN_1702231	79630	C1orf54	chromosome 1 open reading frame 54	1.72	0.016
ILMN_1666503	27147	DENND2A	DENN/MADD domain containing 2A	1.66	0.016
ILMN_1687921	339123	JMJD8	jumonji domain containing 8	1.52	0.017
ILMN_1722622	9332	CD163	CD163 molecule	1.85	0.017
ILMN_1732799	947	CD34	CD34 molecule	1.53	0.018
ILMN_1782419	2791	GNG11	guanine nucleotide binding protein (G protein), gamma 11	1.68	0.018
ILMN_1699574	8829	NRP1	neuropilin 1	1.61	0.019
ILMN_1763568	84287	ZDHHC16	zinc finger, DHHC-type containing 16	1.55	0.019
ILMN_2379599	9332	CD163	CD163 molecule	1.79	0.019
ILMN_1773389	5360	PLTP	phospholipid transfer protein	1.75	0.019
ILMN_1791447	6387	CXCL12	chemokine (C-X-C motif) ligand 12	1.54	0.019
ILMN_1670672	140738	TMEM37	transmembrane protein 37	1.65	0.020
ILMN_1660114	22915	MMRN1	multimerin 1	1.62	0.020
ILMN_1666471	27089	UQCRQ	ubiquinol-cytochrome c reductase, complex III subunit VII, 9.5kDa	1.54	0.021
ILMN_2366391	5052	PRDX1	peroxiredoxin 1	1.60	0.021
ILMN_1784641	4696	NDUFA3	NADH dehydrogenase (ubiquinone) 1 alpha subcomplex, 3, 9kDa	1.51	0.021
ILMN_1795183	6035	RNASE1	ribonuclease, RNase A family, 1 (pancreatic)	1.65	0.022
ILMN_1812968	54345	SOX18	SRY (sex determining region Y)-box 18	1.65	0.022
ILMN_2281810	5672	PSG4	pregnancy specific beta-1-glycoprotein 4	1.88	0.022
ILMN_1686623	1436	CSF1R	colony stimulating factor 1 receptor	1.71	0.023
ILMN_2091347	3417	IDH1	isocitrate dehydrogenase 1 (NADP+), soluble	1.54	0.023
ILMN_1808114	10894	LYVE1	lymphatic vessel endothelial hyaluronan receptor 1	1.59	0.024
ILMN_2128428	1601	DAB2	disabled homolog 2, mitogen-responsive phosphoprotein (Drosophila)	1.67	0.024
ILMN_1693530	5671	PSG3	pregnancy specific beta-1-glycoprotein 3	2.43	0.024
ILMN_1722713	2192	FBLN1	fibulin 1	1.91	0.024
ILMN_1804277	161742	SPRED1	sprouty-related, EVH1 domain containing 1	1.62	0.024
ILMN_1718063	3988	LIPA	lipase A, lysosomal acid, cholesterol esterase	1.63	0.025
ILMN_1728734	5673	PSG5	pregnancy specific beta-1-glycoprotein 5	1.66	0.025
ILMN_1668092	90952	ESAM	endothelial cell adhesion molecule	1.81	0.025
ILMN_1757351	6278	S100A7	S100 calcium binding protein A7	1.71	0.025
ILMN_1657862	191	AHCY	adenosylhomocysteinase	1.68	0.025
ILMN_2341229	947	CD34	CD34 molecule	1.72	0.025
ILMN_1672611	1009	CDH11	cadherin 11, type 2, OB-cadherin (osteoblast)	2.02	0.026
ILMN_1679838	51186	WBP5	WW domain binding protein 5	1.55	0.027
ILMN_2139970	220	ALDH1A3	aldehyde dehydrogenase 1 family, member A3	2.11	0.027
ILMN_2363658	7837	PXDN	peroxidasin homolog (Drosophila)	1.63	0.027
ILMN_3243471	10330	CNPY2	canopy 2 homolog (zebrafish)	1.62	0.028
ILMN_1723684	2532	DARC	Duffy blood group, chemokine receptor	1.74	0.029
ILMN_1681949	5156	PDGFRA	platelet-derived growth factor receptor, alpha polypeptide	1.63	0.029
ILMN_1672536	2192	FBLN1	fibulin 1	2.63	0.029
ILMN_2117330	4708	NDUFB2	NADH dehydrogenase (ubiquinone) 1 beta subcomplex, 2, 8kDa	1.60	0.030
ILMN_3307791	388650	FAM69A	family with sequence similarity 69, member A	1.59	0.030
ILMN_2062701	2619	GAS1	growth arrest-specific 1	2.04	0.031
ILMN_1729188	57817	HAMP	hepcidin antimicrobial peptide	1.85	0.031
ILMN_1663640	4128	MAOA	monoamine oxidase A	1.71	0.031
ILMN_1687301	1462	VCAN	versican	1.61	0.031
ILMN_1774207	285	ANGPT2	angiopoietin 2	1.71	0.032
ILMN_1752728	2517	FUCA1	fucosidase, alpha-L- 1, tissue	1.63	0.033
ILMN_1728197	7122	CLDN5	claudin 5	1.61	0.034
ILMN_1748473	55303	GIMAP4	GTPase, IMAP family member 4	1.55	0.034
ILMN_2309615	5675	PSG6	pregnancy specific beta-1-glycoprotein 6	1.93	0.034
ILMN_1700541	2192	FBLN1	fibulin 1	2.27	0.034
ILMN_2366388	5052	PRDX1	peroxiredoxin 1	1.54	0.035
ILMN_1717163	2162	F13A1	coagulation factor XIII, A1 polypeptide	1.65	0.035
ILMN_1654151	1345	COX6C	cytochrome c oxidase subunit VIc	1.60	0.035
ILMN_2086470	5156	PDGFRA	platelet-derived growth factor receptor, alpha polypeptide	1.68	0.038
ILMN_1741133	4830	NME1	NME/NM23 nucleoside diphosphate kinase 1	1.51	0.039
ILMN_1815057	5159	PDGFRB	platelet-derived growth factor receptor, beta polypeptide	1.97	0.039
ILMN_1772910	2619	GAS1	growth arrest-specific 1	3.08	0.039
ILMN_1696360	1508	CTSB	cathepsin B	1.50	0.040
ILMN_1791576	22856	CHSY1	chondroitin sulfate synthase 1	1.59	0.041
ILMN_1691572	7263	TST	thiosulfate sulfurtransferase (rhodanese)	1.55	0.041
ILMN_1713807	57134	MAN1C1	mannosidase, alpha, class 1C, member 1	1.53	0.042
ILMN_1717262	10544	PROCR	protein C receptor, endothelial	1.54	0.042
ILMN_1651950	8460	TPST1	tyrosylprotein sulfotransferase 1	1.66	0.042
ILMN_2333670	6035	RNASE1	ribonuclease, RNase A family, 1 (pancreatic)	1.81	0.043
ILMN_1670899	2201	FBN2	fibrillin 2	1.71	0.043
ILMN_1751851	51816	CECR1	cat eye syndrome chromosome region, candidate 1	1.50	0.043
ILMN_1810852	3915	LAMC1	laminin, gamma 1 (formerly LAMB2)	1.59	0.043
ILMN_1789196	7169	TPM2	tropomyosin 2 (beta)	1.79	0.044
ILMN_1721035	64231	MS4A6A	membrane-spanning 4-domains, subfamily A, member 6A	1.59	0.046
ILMN_2400935	6876	TAGLN	transgelin	1.56	0.047
ILMN_1752755	7450	VWF	von Willebrand factor	2.35	0.047
ILMN_1801776	5678	PSG9	pregnancy specific beta-1-glycoprotein 9	1.72	0.049
ILMN_1764483	5670	PSG2	pregnancy specific beta-1-glycoprotein 2	1.83	0.049

^a-^ Entrez gene identifier;

^b-^ Symbol- taken from the gene database which corresponds to the official Human Genome Organization Gene Nomenclature Committee symbols

^c-^ Fold change (the number of times the average expression level in the chorioamniotic membranes of the neurocognitive impairment group differs from the average expression level in the normal comparison group

**Table 4:** Underexpressed Illumina probes (N = 28) in the neurocognitive impairment group compared to the no impairment group.

Illumina Probe ID	ENTREZ^a^	SYMBOL^b^	Gene Name	Fold Change^c^	P-Value
ILMN_2188119	339231	ARL16	ADP-ribosylation factor-like 16	1.58	0.001
ILMN_2150654	65249	ZSWIM4	zinc finger, SWIM-type containing 4	1.53	0.001
ILMN_2415748	26118	WSB1	WD repeat and SOCS box containing 1	1.53	0.002
ILMN_2227495	256051	ZNF549	zinc finger protein 549	1.52	0.003
ILMN_1735014	1316	KLF6	Kruppel-like factor 6	1.53	0.005
ILMN_2049364	151194	METTL21A	methyltransferase like 21A	1.53	0.007
ILMN_1737406	1316	KLF6	Kruppel-like factor 6	1.58	0.009
ILMN_1723486	3099	HK2	hexokinase 2	1.85	0.015
ILMN_1659990	29923	HILPDA	hypoxia inducible lipid droplet-associated	1.88	0.016
ILMN_1721349	84061	MAGT1	magnesium transporter 1	1.57	0.016
ILMN_1678833	1230	CCR1	chemokine (C-C motif) receptor 1	1.55	0.016
ILMN_2122511	147372	CCBE1	collagen and calcium binding EGF domains 1	1.50	0.017
ILMN_1680987	9421	HAND1	heart and neural crest derivatives expressed 1	1.95	0.021
ILMN_1787266	6690	SPINK1	serine peptidase inhibitor, Kazal type 1	1.71	0.021
ILMN_2261076	4739	NEDD9	neural precursor cell expressed, developmentally down-regulated 9	1.54	0.022
ILMN_1680313	6810	STX4	syntaxin 4	1.54	0.023
ILMN_1811489	9943	OXSR1	oxidative-stress responsive 1	1.54	0.026
ILMN_1768534	8553	BHLHE40	basic helix-loop-helix family, member e40	1.79	0.028
ILMN_1717877	10625	IVNS1ABP	influenza virus NS1A binding protein	1.76	0.030
ILMN_2397750	10625	IVNS1ABP	influenza virus NS1A binding protein	1.83	0.033
ILMN_2188333	969	CD69	CD69 molecule	1.87	0.034
ILMN_1651365	79413	ZBED2	zinc finger, BED-type containing 2	1.56	0.034
ILMN_1713829	9536	PTGES	prostaglandin E synthase	1.51	0.035
ILMN_1719695	64332	NFKBIZ	nuclear factor of kappa light polypeptide gene enhancer in B-cells inhibitor, zeta	1.78	0.036
ILMN_1720158	2114	ETS2	v-ets erythroblastosis virus E26 oncogene homolog 2 (avian)	1.51	0.043
ILMN_2197128	130497	OSR1	odd-skipped related 1 (Drosophila)	1.54	0.043
ILMN_1702691	7128	TNFAIP3	tumor necrosis factor, alpha-induced protein 3	1.88	0.044
ILMN_1682775	1906	EDN1	endothelin 1	1.82	0.044

^a-^ Entrez gene identification;

^b-^ Symbol- taken from the gene database which corresponds to the official Human Genome Organization Gene Nomenclature Committee symbols

^c-^ Fold change (the number of times the average expression level in the chorioamniotic membranes of the neurocognitive impairment group differs from the average expression level in the normal comparison group

**Table 5:** Biological processes enriched for genes differentially expressed between the neurocognitive impairment and no impairment groups.

Biological Process Category	Differentially expressed genes/ total genes in GO^a^ term	Odds Ratio of enrichment	FDR^b^ adjusted p-value
Multicellular organismal process	60/4070	3.0	0.0001
Metanephric glomerulus vasculature development	4/8	112.6	0.0003
Localization of cell	21/759	3.6	0.0018
Renal system vasculature development	4/14	45.9	0.0019
Positive regulation of cellular component movement	10/212	5.8	0.0063
Female pregnancy	6/71	10.7	0.0094
Lymphangiogenesis	3/10	47.8	0.014
Positive regulation of monocyte chemotaxis	3/10	47.8	0.014
Retina vasculature development in camera-type eye	3/11	41.8	0.017
Regulation of cell motility	12/370	4.0	0.019
Positive regulation of cell proliferation by VEGF-activated platelet derived growth factor receptor signaling pathway	2/3	220.9	0.024
Metanephric glomerulus morphogenesis	2/3	220.9	0.024
Metanephric glomerular capillary formation	2/3	220.9	0.024
Metanephric nephron development	4/34	15.0	0.024
Positive regulation of chemotaxis	5/69	8.9	0.035
Positive regulation of ERK1 and ERK2 cascade	5/70	8.7	0.037
Lymphatic endothelial cell differentiation	2/4	110.4	0.037
Glomerular endothelium development	2/4	110.4	0.037
Middle ear morphogenesis	3/18	22.3	0.039

a GO: Gene ontology;

b FDR: False Discovery Rate.

**Table 6:** Molecular functions enriched for genes differentially expressed between the neurocognitive impairment and no impairment groups.

Molecular Function Category	Differentially expressed genes/ total genes in GO^a^ term	Odds Ratio	FDR^b^ adjusted p-value
Vascular endothelial growth factor-activated receptor activity	3/5	173.1	0.0018
Vascular endothelial growth factor binding	2/3	228.6	0.032
Cytokine binding	4/40	13.0	0.037

a GO: Gene ontology

b FDR: False Discovery Rate.

### qRT-PCR results

48 out of 90 selected differentially expressed genes were confirmed by qRT-PCR, including genes implicated in neuroinflammation, neurodegeneration and cognitive disorders (e.g., up regulation of *SEPP1*, *APOE*, *DAB2*, *CD163*, *CXCL12*, *VWF* and down-regulation of *HAND1*, *OSR1*) (p<0.05). (**Table 7**) In addition to genes previously described as playing a critical role in cognition, genes involved in neuronal differentiation, signaling, vascular permeability and cellular metabolism also were identified.

**Table 7:** Comparison of qRT-PCR and microarray analysis of select genes, with direction of change denoting change in group with neurocognitive impairment.

Gene Symbol	Gene Name	Fold change qRT-PCR^a^	P-value qRT-PCR	Fold change microarray^a^	P-value microarray
HAND1	heart and neural crest derivatives expressed 1	-5.18	0.000	-1.95	0.021
SPRED1	sprouty-related, EVH1 domain containing 1	1.81	0.001	1.62	0.024
LGMN	Legumain	2.00	0.001	1.81	0.010
ADAMTS9	ADAM metallopeptidase with thrombospondin type 1 motif, 9	2.79	0.001	1.60	0.010
NRP1	neuropilin 1	2.09	0.002	1.61	0.019
VSIG4	V-set and immunoglobulin domain containing 4	2.27	0.003	1.60	0.002
ALDH1A3	aldehyde dehydrogenase 1 family, member A3	2.17	0.003	2.11	0.027
CD163	CD163 molecule	2.28	0.003	1.85	0.017
ANGPT2	angiopoietin 2	3.02	0.006	1.71	0.032
CD34	CD34 molecule	3.83	0.007	1.53	0.018
OSR1	odd-skipped related 1 (Drosophila)	-2.90	0.007	-1.54	0.043
PECAM1	platelet/endothelial cell adhesion molecule 1	1.78	0.009	1.51	0.006
CTSB	cathepsin B	1.48	0.009	1.69	0.012
WSB1	WD repeat and SOCS box containing 1	-1.41	0.009	-1.53	0.002
UCP2	uncoupling protein 2 (mitochondrial, proton carrier)	1.71	0.010	1.50	0.012
SEPP1	selenoprotein P, plasma, 1	2.49	0.010	2.58	0.009
LDHB	lactate dehydrogenase B	1.69	0.011	1.64	0.007
SLCO2B1	solute carrier organic anion transporter family, member 2B1	2.30	0.011	1.61	0.014
LY6G6C	lymphocyte antigen 6 complex, locus G6C	-2.52	0.012	-1.34	0.058
VWF	von Willebrand factor	3.52	0.013	2.35	0.047
VCAN	versican	1.69	0.013	1.61	0.031
COLEC12	collectin sub-family member 12	1.89	0.015	1.77	0.014
LIPA	lipase A, lysosomal acid, cholesterol esterase	1.71	0.016	1.63	0.025
CXCL12	chemokine (C-X-C motif) ligand 12	2.83	0.017	1.54	0.019
APOE	apolipoprotein E	2.54	0.017	2.46	0.056
GPD1L	glycerol-3-phosphate dehydrogenase 1-like	1.85	0.018	1.54	0.013
MS4A6A	membrane-spanning 4-domains, subfamily A, member 6A	1.82	0.019	1.79	0.006
CSF1R	colony stimulating factor 1 receptor	1.71	0.019	1.71	0.023
MMRN1	multimerin 1	3.79	0.021	1.62	0.020
LYVE1	lymphatic vessel endothelial hyaluronan receptor 1	2.03	0.021	1.59	0.024
RDH11	retinol dehydrogenase 11 (all-trans/9-cis/11-cis)	1.30	0.023	1.52	0.001
PDGFRA	platelet-derived growth factor receptor, alpha polypeptide	1.82	0.023	1.63	0.029
OLFML2B	olfactomedin-like 2B	2.07	0.023	1.63	0.005
PDGFRB	platelet-derived growth factor receptor, beta polypeptide	1.97	0.026	1.97	0.039
DAB2	disabled homolog 2, mitogen-responsive phosphoprotein (Drosophila)	1.52	0.029	1.78	0.015
C1orf54	chromosome 1 open reading frame 54	1.63	0.029	1.72	0.016
STX4	syntaxin 4	-1.43	0.029	-1.54	0.023
FAM69A	family with sequence similarity 69, member A	1.99	0.029	1.59	0.030
CHI3L2	chitinase 3-like 2	1.72	0.032	1.83	0.057
PROCR	protein C receptor, endothelial	1.80	0.034	1.54	0.042
MAOA	monoamine oxidase A	1.44	0.037	1.71	0.031
DARC	Duffy blood group, chemokine receptor	2.82	0.037	1.74	0.029
FBLN1	fibulin 1	1.95	0.041	1.91	0.024
CDH11	cadherin 11, type 2, OB-cadherin (osteoblast)	1.74	0.044	2.02	0.026
NEDD9	neural precursor cell expressed, developmentally down-regulated 9	-1.45	0.044	-1.54	0.022
OXSR1	oxidative-stress responsive 1	-1.39	0.046	-1.54	0.026
ESAM	endothelial cell adhesion molecule	1.82	0.047	1.81	0.025
EDN1	endothelin 1	-1.57	0.049	-1.82	0.044

^a^ Fold change- the number of times the average expression level in the chorioamniotic membranes of the neurocognitive impairment group differs from the average expression level in the normal comparison group; positive values (no sign) denote increased expression (up-regulation) in the neurocognitive impairment group, and negative values (minus sign) indicate decreased expression (down-regulation) in the neurocognitive impairment group.

### Gene-based classifier

A gene-based classifier was developed using qRT-PCR measured expression data from 14 controls and 14 cases using a vetted pipeline that automatically determines the appropriate number of markers using an internal cross-validation procedure. The candidate predictors considered as inputs in this pipeline were the 90 genes selected based on the microarray data. Instead of using the Ct value of a reference gene (e.g., GAPDH) as an internal normalizer for each sample, we searched for the best normalizer among the 90 candidate genes. In doing so, we attempted to accomplish three goals: 1) convert the Ct values into a platform-independent measure (the ratio of the expression of two genes), 2) explore potential gene interactions in predicting neurocognitive outcome, and 3) avoid inclusion of a gene that brings no discrimination between phenotypes in the model. Our methodology identified the ratios *OSR1/VWF* and *HAND1/VWF* as providing the best cross-validated performance on the training data when used as the inputs in a linear discriminant analysis model (70% average sensitivity over three specificity values: 80, 85, and 90%). Other classifiers such as quadratic discriminant analysis and support vector machines had similar, yet lower performance (62 and 68% respectively), and hence the linear discriminant analysis model was retained as the final model.

**Fig 3:**
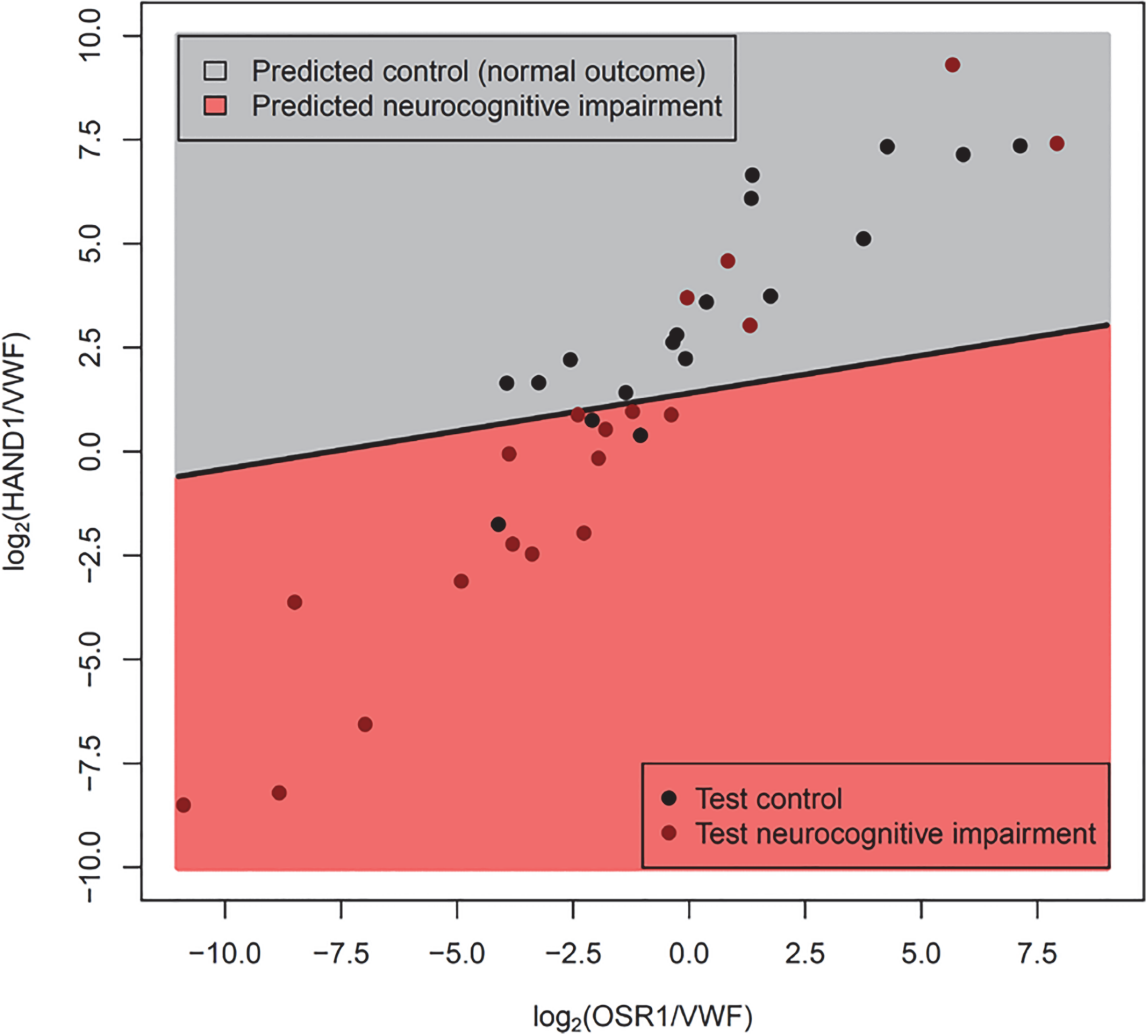
Gene expression-based classifier for neurocognitive impairment using OSR1/VWF and HAND1/VWF expression ratios. The Fig. shows the linear discriminant model (see oblique black line) built using qRT-PCR measured expression data from the training set. Since −Ct values are surrogate for log2 gene abundance, differences in −Ct values of two genes is equivalent to their log_2_ expression ratios. Data are represented as log2 expression ratios (y-axis: −Ct _HAND1_ +Ct _VWF_; x-axis: −Ct _OSR1_ +Ct _VWF_). The dots represent data from patients from the test set. The model was tuned on the training data to yield a specificity of ∼85%. The actual performance on the test set was sensitivity of 74%, at specificity of 83%.

**Fig 4:**
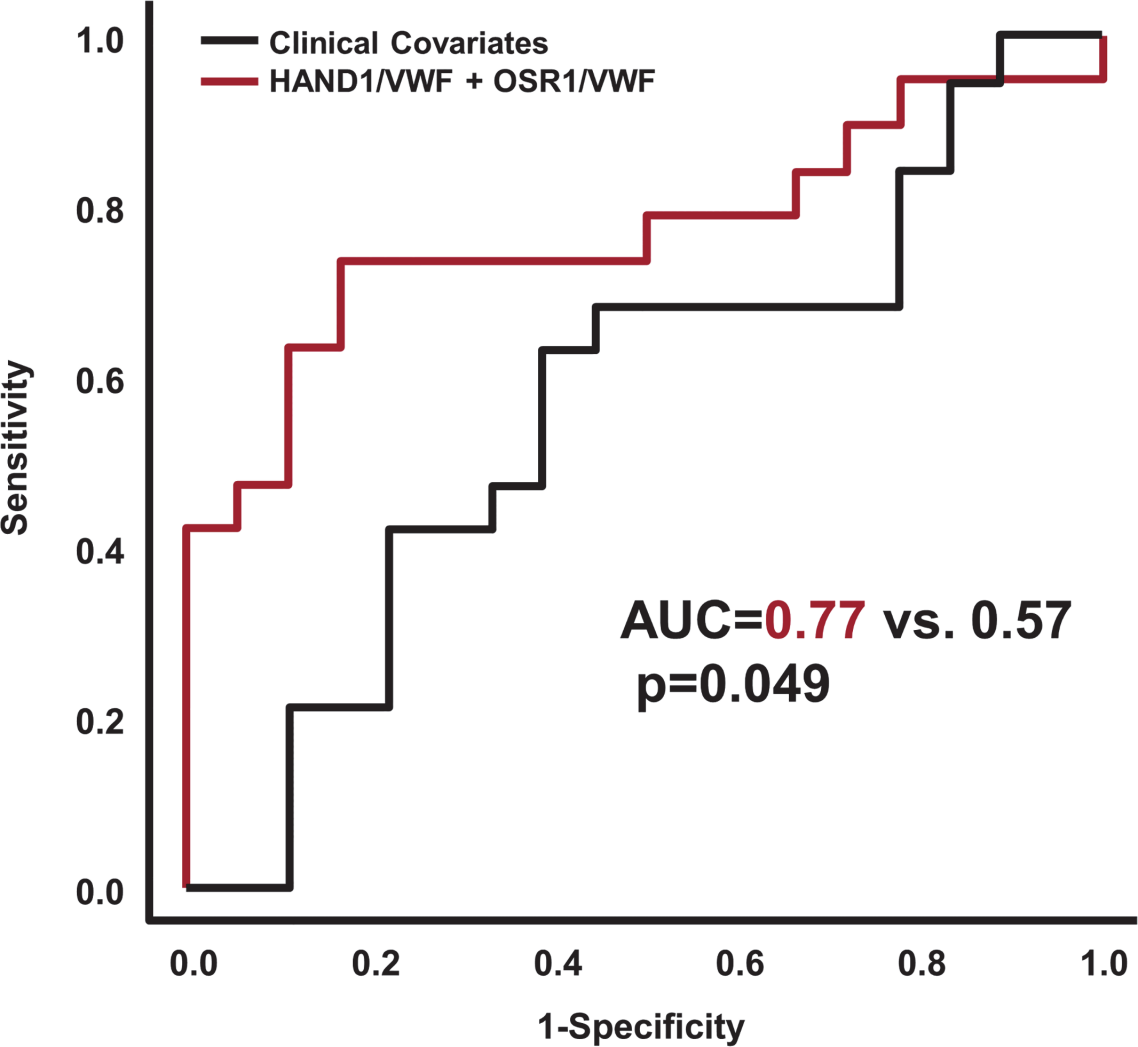
Performance of gene expression-based classifier on test set compared with a model using clinical covariates. When compared with clinical covariates available at the time of birth, the molecular prediction model had superior Area under the Receiver Operating Characteristic curve (AUC 0.77 vs 0.57, p = 0.049). Clinical covariates included the following: gestational age, gender, small for gestational age status, 5-minute Apgar score, labor and chorioamnionitis. The p-value for the difference between the two ROC curves was obtained using a bootstrap based method implemented in the pROC package in R (http://www.-r-project.org).

This model had a sensitivity of 74% and a specificity of 83% when applied to a new set of patients (18 Controls and 19 Disease, as 1 Control was discarded due to PCR failures for multiple genes) (See **Fig. 3**). Although most of the misclassified samples are close to the decision boundary (black line in **Fig. 3**), two of the misclassified cases with neurocognitive impairment had very high *OSR1/VWF* and *HAND1/VWF* gene ratios; these cases had multiple post-natal complications (specifically, severe bronchopulmonary dysplasia, necrotizing enterocolitis and post-natal sepsis) often associated with neurodevelopmental impairment. When compared with clinical covariates available at the time of birth, the molecular prediction model had superior Area Under the Receiver Operating Characteristic curve (AUC 0.77 vs 0.57, p = 0.049) as determined by a bootstrap based test implemented in the *pROC* package [61]. (**Fig. 4**).

As suggested by an anonymous reviewer, we also examined the effect of gestational age (GA) at delivery on the quality of predictions of the gene based classifier. The subjects in the test dataset were divided based on gestational age at delivery: extremely preterm (GA from 23.9 to 26.9) and very preterm (GA from 27.0 to 32.1), where the cut-off point of 27.0 weeks was the median of gestational age at delivery in the test set. Although the point estimate of the area under the receiver operating characteristic curve (AUC) was higher for the extremely preterm group (AUC = 82%) compared to the one for the very preterm group (AUC = 70%) the difference was not significant, and including GA at delivery as a covariate in the LDA model (either as a main effect or interaction with the two gene based predictors) did not improve the classifier prediction.

## Discussion

The principal findings of our study were: **1**) the chorioamniotic membrane transcriptome of preterm neonates with cognitive impairment differed significantly from that of neonates with normal neurodevelopment; **2**) Gene ontology analysis indicated enrichment of 19 biological processes (e.g., positive regulation of cell proliferation by VEGF-activated platelet derived growth factor receptor signaling pathway, etc.) and 3 molecular functions (cytokine binding, vascular-endothelial growth factor receptor activity and vascular-endothelial growth factor receptor binding); **3**) **PADOG** identified 4 significantly enriched KEGG pathways: oxidative phosphorylation, Parkinson’s disease, Alzheimer’s disease and Huntington’s disease (q-value <0.1); **4**) 48 out of 90 selected differentially expressed genes were confirmed by qRT-PCR, including genes implicated in energy metabolism, neuronal differentiation, signaling, vascular permeability and response to injury (e.g., up-regulation of *SEPP1*, *APOE*, *DAB2*, *CD163*, *CXCL12*, *VWF*; down-regulation of *HAND1*, *OSR1*) (p<0.05 and fold change >1.5); and **5**) cases with neurocognitive impairment at 18–24 months could be identified at birth apart from gestational age-matched controls with a sensitivity of 74% at a specificity of 83% using a molecular signature determined in an independent sample (using ratios of OSR1/VWF and HAND1/VWF in the chorioamniotic membranes). These findings support the view that disturbances in oxidative metabolism, synaptic signaling and response to injury identified at birth, contribute elevated risk of neurocognitive impairment assessed in early childhood.

### Molecular marker odd-skipped related 1 (OSR1) gene, downregulated in cases

The Osr1 gene encodes a zinc finger transcription factor that modulates embryonic patterning and morphogenesis as a pair-rule gene; mutations in this gene in *Drosophila* lead to a loss of odd segments during embryogenesis, thus the name odd-skip related [62]. Human Osr1 consists of three exons located on chromosome 2p24 [63], a region recently implicated as a candidate risk susceptibility locus for autism spectrum disorder [64, 65].

The Osr1 transcription factor encoded by this gene may have functional protein-protein interactions with Oxidative stress responsive 1 kinase (OXSR1) [66, 67]. This kinase phosphorylates the Na^+^-K^+^-2Cl^—^(NKCC1) co-transporter, responsible for the high intra-cellular chloride concentration of immature neurons contributing to their depolarization (and excitation) on GABA binding [68]. GABA, the main inhibitory neurotransmitter in adults, serves as an excitatory neurotransmitter during fetal and early neonatal life [69, 70]. GABA excitation stimulates giant depolarizing potentials which mediate activity-dependent stimulation of neuronal growth, migration, synapse formation and development of functional brain networks. Dysregulation of early GABAergic signaling may lead to aberrant neuronal circuitry and impaired cognitive functioning and is implicated in autism. Interestingly, the Osr1 transcription factor plays a role in regulating renal development, a tissue rich in chloride transporters and vascular tight junctions [71, 72].

### Molecular marker heart and neural crest derivatives expressed 1 (HAND1), downregulated in cases

Heart and neural crest derivatives expressed 1 (Hand1, also known as eHand) encodes a Twist-family basic helix-loop-helix transcription factor that plays a role in placentation, trophoblast differentiation [73–76], fetal cardiac [77, 78] and fetal sympathetic nervous system development [79–81]: tissues all known for their high energy demands and sensitivity to hypoxia signaling. Until recently, understanding of Hand1 function remained poor due to the early embryonic lethality of Hand1-knockout animals [80, 82]. Null and hypomorphic mouse models revealed defects in extraembryonic mesoderm and trophoblast giant cells associated with reduced expression of Placental Lactogen I (*Pl1*), a placental hormone important for maintenance of the corpus luteum and normal progesterone levels [82, 83]. In addition, a developmental arrest in yolk sac vasculogenesis was revealed, followed by increased expression of angiogenic growth factors [75]. Despite these findings, Hand1 deficient embryos had down-regulated HIF-1α mRNA expression.

Recently, Breckenridge et al reported that Hand1 was induced by hypoxia and HIF-1α binding upstream of the Hand1 transcriptional start site in mice; additionally, Hand1 expression was associated with decreased oxygen consumption via down-regulation of lipid metabolism and uptake in fetal and adult cardiomyocytes [84]. Thus, Hand1 appears to mediate mitochondrial energy generation and the fetal to neonatal switch from glycolytic to oxidative metabolism [84]. Decreased Hand1 expression is associated with up-regulation of genes encoding proteins involved in lipid uptake and mitochondrial β-oxidation. Whether Hand1 plays a similar role in the central nervous system (CNS) remains to be elucidated. In the CNS, Hand1 is expressed in sympathetic neurons [80] and regulates neuronal sympathetic survival and differentiation along with Hand2 and homeodomain transcription factor Phox2b [81].

### Apolipoprotein E (APOE) upregulated in cases

We identified several other genes involved in neuronal differentiation, lipid uptake and CNS signaling. The Apolipoprotein E gene (*APOE*) encodes a multifunctional glycoprotein that transports lipids and cholesterol in the plasma and CNS by binding to low-density lipoprotein receptors [85]. APOE/lipoprotein particles are produced by astrocytes [86] and to some extent microglia [87]. With brain injury, APOE expression is up-regulated. APOE interacts with cytokines and alters macrophage function, suppresses T cell proliferation, up-regulates platelet nitric oxide production and increases lipid antigen presentation by CD1 molecules; it also maintains the integrity of the blood brain and blood nerve barriers [88]. In the brain, APOE binds to the very low-density lipoprotein receptor (VLDLR) and APOE receptor 2 (APOER2), the two main reelin signaling receptors. APOE can significantly inhibit reelin binding and subsequent phosphorylation of the adapter molecule disabled 1 protein (dab1) which initiates the intracellular transduction of reelin signaling [89]. Mutations in the *RELN* and *DAB1* genes that disrupt reelin signaling are associated with cerebellar hypoplasia and severe abnormalities in neuronal organization and migration [90–92]; mutations in either VLDLR or APOER2 in isolation result in more subtle defects in cell positioning, synapse and dendritic spine formation [93]. APOE genotype/expression has been linked to human neurocognitive and neuroinflammatory disorders (e.g., Alzheimer’s disease [94], Parkinson’s disease [95–97] multiple sclerosis [98] and HIV disease progression [99]). Preliminary evidence also suggests an association between APOE gene expression and altered brain structure at birth (alterations in regional cortical brain volumes) [100].

### Selenoprotein P, plasma 1 (SEPP1) upregulated in cases

The selenoprotein P, Plasma 1 (*Sepp1*) gene encodes a selenium rich extracellular protein involved in selenium transport and antioxidant defense mechanisms in the brain [101]. In the setting of brain injury, Sepp1 is up-regulated and secreted by astrocytes [102]; it is then taken up by neurons via the APOER2 which also binds reelin [103–105]. Interruption of the reelin signaling pathway may have devastating effects on brain development as previously noted. Increased Sepp1 expression is reported in neuroinflammatory disorders associated with impaired cognition. Bellinger *et al* reported increased expression in post-mortem Parkinson’s disease brain tissues (in Lewy bodies and the substantia nigra, relative to neuron count) [106] and in Alzheimer’s disease (in amyloid beta plaques and neurofibrillary tangles) [107].

### Disabled homolog 2, mitogen responsive phosphoprotein (DAB2) upregulated in cases

The disabled homolog 2, mitogen responsive phosphoprotein (Drosophila) gene (*Dab2*) encodes disabled protein 2. Murine disabled-2 (initially termed p96) was isolated as a 96 kD phosphoprotein involved in macrophage signaling via colony stimulating factor-1 [108]. Sequence homology suggested that p96 was an ortholog of the Drosophila disabled gene[109] and this was the origin for the names of neuronally expressed Dab1 and the more broadly expressed Dab2. Recently, Dab2 expression was shown to be up-regulated by macrophages and astrocytes in various CNS injury models (e.g., cryoinjury [110] and autoimmune encephalomyelitis [111]). In humans, Dab2 was up-regulated in a microarray study of autopsy specimens in multiple sclerosis lesions.

Dab2 has several functions. Similar to Dab1, it serves as an intracellular signaling protein that mediates cell organization and positioning by regulating Src activity and the mitogen-activated protein kinase signaling pathway [112]. Dab2 also is involved in lipid receptor endocytosis (including APOER2) [113] and neurotransmitter release [114]. In addition, Dab2 interacts with the N-terminal domain of Dab2 interacting protein [115]; this protein regulates dendrite development, synapse formation and neuronal migration in the cerebellum and developing cortex [116, 117].

### Other genes involved in vascular endothelial function and response to injury: von Willebrand factor (VWF), Cluster of Differentiation 163 (CD-163) and C-X-C motif chemokine 12 (CXCL-12) upregulated in cases compared with controls

*VWF* gene is upregulated in cases. The von Willebrand factor gene encodes a large plasma glycoprotein that is synthesized by vascular endothelial cells and megakaryocytes in response to endothelial injury. It plays a central role in platelet adhesion, activation and thrombin generation [118]. Compared with older individuals, preterm infants and fetuses have higher concentrations and larger multimers of VWF [118–120]. By combining the signal from this up-regulated gene with that of the OSR1 and HAND1 genes that are down-regulated in disease, we obtain two dimensionless variables (OSR1/VWF and HAND1/VWF) that 1) differ to a greater extent between cases and controls, 2) are platform-independent and 3) lead to a gene classifier that is more cost-effective as compared to one that uses a standard gene normalizer (e.g., GAPDH) with no discriminatory power.

*CD 163* gene is upregulated in cases. The protein encoded by the CD163 gene is a member of the scavenger receptor cysteine-rich (SRCR) superfamily that is expressed by monocytes/macrophages. In the CNS, CD163 is localized to perivascular macrophages and microglia. It is thought to function as an innate immune sensor for bacteria altering local immune responsiveness and an acute phase-regulated receptor involved in the clearance and endocytosis of hemoglobin/haptoglobin complexes protecting tissues from free hemoglobin-mediated oxidative damage [121]. CD163 expression is regulated by both proinflammatory and anti-inflammatory mediators (suppressed by lipopolysaccharide, interferon-gamma and tumor necrosis factor alpha and strongly up-regulated by IL-6 and IL-10)[122]. Increased CD163 expression has been linked to neuroinflammatory disease states such as multiple sclerosis[123], Alzheimer’s disease[124]) HIV-associated neurocognitive disorders[125] and schizophrenia[126].

*CXCL12* gene is upregulated in cases. The C-X-C motif chemokine 12 gene also known as stromal cell-derived factor 1 alpha (SDF-1α) encodes a chemokine protein that binds to chemokine receptor 4 and 7 (G-protein coupled receptors). CXCL12 is induced by proinflammatory stimuli (e.g., lipopolysaccharide, IL1β, TNFα) and has many diverse functions. In the brain, CXCL12 is produced by both neurons and glial cells and is involved in neurogenesis, axonal guidance, neurite outgrowth, modulation of neuronal excitability, neurotransmitter release (particularly GABA release), and neurotransmitter systems cross-talk (e.g., GABA, glutamate, opioids)[127, 128].

CXCL12 also is involved in immune functions (immune surveillance, response to inflammation, leukocyte activation) and vasculogenesis.

### Strengths and limitations

The strengths of our study include (1) the novel study design using transcriptomics of the chorioamniotic membranes, an abundant source of fetal DNA and of fetal stem cells that may be impacted by the intrauterine environment and (2) the use of state-of-the-art analytics to identify perturbed disease pathways and to develop a multi-gene disease classifier. Limitations to be addressed in future studies include the assessment of neurocognitive outcomes at a later time point when follow-up outcomes are deemed more stable, testing of the molecular disease classifier using a second independent sample with the full spectrum of neurocognitive outcomes collected prospectively, and exploration of alternative or complementary biomarkers (additional biological specimens coupled with advanced neuroimaging techniques).

## Conclusion

Impaired brain function in preterm neonates is thought to arise from (1) inflammation and/or hypoxic-ischemic injury to developing preoligodendrocytes and cortical neurons, (2) secondary atrophy after sublethal axonal injury and (3) an arrest or alteration of the developmental trajectory postnatally [129, 130]. We propose that this alteration in the developmental trajectory also arises *prenatally* from stimuli that alter cellular metabolism, neuronal differentiation, signaling, vascular permeability and response to injury. Together, the genes and biological pathways that we have identified provide important preliminary data for the mechanistic processes that may mediate brain injury and aberrant neuronal development in utero. Prospective cohort studies are needed to determine whether this information can be used to identify newborns that will develop neurocognitive impairment in early childhood and might benefit from early intervention or neuro-protective strategies.

## Supporting Information

S1 Table Primers used in qRT-PCR assays with the Biomark system. (DOCX)
