# Supplementary material for: Transcriptomics of Maternal and Fetal Membranes Can Discriminate between Gestational-Age Matched Preterm Neonates with and without Cognitive Impairment Diagnosed at 18–24 Months
Source: PLoS One. 2015 Mar 30;10(3):e0118573. doi: 10.1371/journal.pone.0118573 (PMC4379164; doi:10.1371/journal.pone.0118573)
Supplement: S1 Table — (DOCX) [file pone.0118573.s001.docx]

**Table S1. Primers used in qRT-PCR assays with the Biomark™ system**

| **Gene Symbol^a^** | **Protein name** | **Biomark™ primer** |
| --- | --- | --- |
| ACVRL1 | activin A receptor type II-like 1 | Hs00953798_m1 |
| ADAMTS9 | ADAM metallopeptidase with thrombospondin type 1 motif, 9 | Hs00172025_m1 |
| ADAP2 | ArfGAP with dual PH domains 2 | Hs01106939_m1 |
| ALDH1A3 | aldehyde dehydrogenase 1 family, member A3 | Hs00167476_m1 |
| ANGPT2 | angiopoietin 2 | Hs01048043_m1 |
| APOE | apolipoprotein E | Hs00171168_m1 |
| APOC1 | apolipoprotein C-I | Hs00155790_m1 |
| ARL16 | ADP-ribosylation factor-like 16 | Hs01586770_g1 |
| C1orf54 | chromosome 1 open reading frame 54 | Hs00226199_m1 |
| C4orf48 | chromosome 4 open reading frame 48 | Hs00415607_m1 |
| CCR1 | chemokine (C-C motif) receptor 1 | Hs00174298_m1 |
| CD163 | CD163 molecule | Hs00174705_m1 |
| CD24 | CD24 molecule | Hs00273561_s1 |
| CD34 | CD34 molecule | Hs00990732_m1 |
| CD69 | CD69 molecule | Hs00934033_m1 |
| CDH11 | cadherin 11, type 2, OB-cadherin (osteoblast) | Hs00901475_m1 |
| CHI3L2 | chitinase 3-like 2 | Hs00970220_m1 |
| CLDN5 | claudin 5 | Hs00533949_s1 |
| COLEC12 | collectin sub-family member 12 | Hs00560477_m1 |
| COX6C | cytochrome c oxidase subunit VIc | Hs00269977_m1 |
| CSF1R | colony stimulating factor 1 receptor | Hs00911250_m1 |
| CTSB | cathepsin B | Hs00947433_m1 |
| CXCL12 | chemokine (C-X-C motif) ligand 12 | Hs00171022_m1 |
| DAB2 | disabled homolog 2, mitogen-responsive phosphoprotein (Drosophila) | Hs01120074_m1 |
| DARC | Duffy blood group, chemokine receptor | Hs01011079_s1 |
| DENND2A | DENN/MADD domain containing 2A | Hs01128613_m1 |
| EDN1 | endothelin 1 | Hs00174961_m1 |
| ESAM | endothelial cell adhesion molecule | Hs00332781_m1 |
| FAM69A | family with sequence similarity 69, member A | Hs00961685_m1 |
| FBLN1 | fibulin 1 | Hs00972609_m1 |
| FOLR2 | folate receptor 2 (fetal) | Hs01044732_g1 |
| GAS1 | growth arrest-specific 1 | Hs00266715_s1 |
| GNG11 | guanine nucleotide binding protein (G protein), gamma 11 | Hs00914578_m1 |
| GPD1L | glycerol-3-phosphate dehydrogenase 1-like | Hs00380518_m1 |
| GSTM1 | glutathione S-transferase mu 1 | Hs01683722_gH |
| HAND1 | heart and neural crest derivatives expressed 1 | Hs02330376_s1 |
| HILPDA | hypoxia inducible lipid droplet-associated | Hs00203383_m1 |
| HK2 | hexokinase 2 | Hs00606086_m1 |
| HNRNPAB | heterogeneous nuclear ribonucleoprotein A/B | Hs00954054_g1 |
| LY6G6C | lymphocyte antigen 6 complex, locus G6C | Hs00228938_m1 |
| KLF6 | Kruppel-like factor 6 | Hs00810569_m1 |
| LBP | lipopolysaccharide binding protein | Hs00188074_m1 |
| LDHB | lactate dehydrogenase B | Hs00929956_m1 |
| LGMN | legumain | Hs00271599_m1 |
| LIPA | lipase A, lysosomal acid, cholesterol esterase | Hs01548815_m1 |
| LPAR1 | lysophosphatidic acid receptor 1 | Hs00173500_m1 |
| LYVE1 | lymphatic vessel endothelial hyaluronan receptor 1 | Hs00272659_m1 |
| MAGT1 | magnesium transporter 1 | Hs00259564_m1 |
| MAOA | monoamine oxidase A | Hs00165140_m1 |
| METTL21A | methyltransferase like 21A | Hs00697611_m1 |
| MMRN1 | multimerin 1 | Hs00201182_m1 |
| MS4A6A | membrane-spanning 4-domains, subfamily A, member 6A | Hs00223521_m1 |
| NAPSB | napsin B aspartic peptidase, pseudogene | Hs01383703_g1 |
| NDUFA3 | NADH dehydrogenase (ubiquinone) 1 alpha subcomplex, 3, 9kDa | Hs01547166_g1 |
| NDUFB2 | NADH dehydrogenase (ubiquinone) 1 beta subcomplex, 2, 8kDa | Hs00190006_m1 |
| NEDD9 | neural precursor cell expressed, developmentally down-regulated 9 | Hs00610590_m1 |
| NME1 | NME/NM23 nucleoside diphosphate kinase 1 | Hs02621161_s1 |
| NRP1 | neuropilin 1 | Hs00826128_m1 |
| OLFML2B | olfactomedin-like 2B | Hs00295836_m1 |
| OSR1 | odd-skipped related 1 (Drosophila) | Hs00377071_m1 |
| OXSR1 | oxidative-stress responsive 1 | Hs00178247_m1 |
| PDGFRA | platelet-derived growth factor receptor, alpha polypeptide | Hs00998018_m1 |
| PDGFRB | platelet-derived growth factor receptor, beta polypeptide | Hs01019589_m1 |
| PECAM1 | platelet/endothelial cell adhesion molecule 1 | Hs00169777_m1 |
| PLTP | phospholipid transfer protein | Hs00272126_m1 |
| PRDX1 | peroxiredoxin 1 | Hs00602020_mH |
| PROCR | protein C receptor, endothelial | Hs00941182_m1 |
| PSG3 | pregnancy specific beta-1-glycoprotein 3 | Hs00360732_m1 |
| PXDN | peroxidasin homolog (Drosophila) | Hs00395488_m1 |
| RDH11 | retinol dehydrogenase 11 (all-trans/9-cis/11-cis) | Hs00211283_m1 |
| S100A7 | S100 calcium binding protein A7 | Hs00161488_m1 |
| SEPP1 | selenoprotein P, plasma, 1 | Hs01032845_m1 |
| SIL1 | SIL1 homolog, endoplasmic reticulum chaperone (S. cerevisiae) | Hs00223835_m1 |
| SLC40A1 | solute carrier family 40 (iron-regulated transporter), member 1 | Hs00205888_m1 |
| SLCO2B1 | solute carrier organic anion transporter family, member 2B1 | Hs01030343_m1 |
| SOX18 | SRY (sex determining region Y)-box 18 | Hs00746079_s1 |
| SPRED1 | sprouty-related, EVH1 domain containing 1 | Hs01084559_m1 |
| STX4 | syntaxin 4 | Hs00190266_m1 |
| TAGLN | transgelin | Hs01038777_g1 |
| TFPI | tissue factor pathway inhibitor (lipoprotein-associated coagulation inhibitor) | Hs00196731_m1 |
| TMEM37 | transmembrane protein 37 | Hs01931464_s1 |
| TNFAIP3 | tumor necrosis factor, alpha-induced protein 3 | Hs00234713_m1 |
| UCP2 | uncoupling protein 2 (mitochondrial, proton carrier) | Hs01075227_m1 |
| UQCRQ | ubiquinol-cytochrome c reductase, complex III subunit VII, 9.5kDa | Hs00429571_g1 |
| VCAN | versican | Hs00171642_m1 |
| VSIG4 | V-set and immunoglobulin domain containing 4 | Hs01075227_m1 |
| VWF | von Willebrand factor | Hs01109446_m1 |
| WSB1 | WD repeat and SOCS box containing 1 | Hs00373204_m1 |
| ZNF549 | zinc finger protein 549 | Hs00938294_m1 |
| ZSWIM4 | zinc finger, SWIM-type containing 4 | Hs00397653_m1 |
|  |  |  |
| GAPDH | glyceraldehyde-3-phosphate dehydrogenase | Hs99999905_m1 |
| RPLPO | ribosomal protein, large, P0 | Hs99999902_m1 |
| ACTIN | actin, beta | 4352668 |

1. Gene symbol- corresponds to the official Human Genome Organization Gene Nomenclature Committee symbols
